# Baseline Peritoneal Membrane Transport Characteristics Are Associated with Peritonitis Risk in Incident Peritoneal Dialysis Patients

**DOI:** 10.3390/membranes12030276

**Published:** 2022-02-28

**Authors:** Yi-Hsin Chou, Yung-Tai Chen, Jinn-Yang Chen, Der-Cherng Tarng, Chih-Ching Lin, Szu-Yuan Li

**Affiliations:** 1 Division of Nephrology, Department of Medicine, Taipei Veterans General Hospital, Taipei 11217, Taiwan; yhchou7@vghtpe.gov.tw (Y.-H.C.); jychen@vghtpe.gov.tw (J.-Y.C.); dctarng@vghtpe.gov.tw (D.-C.T.); lincc2@vghtpe.gov.tw (C.-C.L.); 2 Department of Medicine, Taipei City Hospital Heping Fuyou Branch, Taipei 11217, Taiwan; ytchen0117@gmail.com; 3 Institute of Clinical Medicine, National Yang-Ming Chiao-Tung University, Taipei 11217, Taiwan; 4 School of Medicine, National Yang-Ming Chiao-Tung University, Taipei 11217, Taiwan; 5 Department and Institute of Physiology, National Yang-Ming University, Taipei 11217, Taiwan

**Keywords:** peritoneal dialysis, peritonitis, peritoneal equilibration test

## Abstract

The peritoneal equilibration test (PET) is a semi-quantitative measurement that characterizes the rate of transfer of solutes and the water transfer rate across the peritoneum in patients treated with peritoneal dialysis (PD). The results of the PET are used to maximize daily peritoneal ultrafiltration and solute clearances. Previous studies have shown that high transport status is associated with ultrafiltration failure, malnutrition, and reduced survival; however, the way in which peritoneum transport characteristics affect peritonitis risk is unknown. In the current cohort study, we recruited 898 incident-PD patients and used intention-to-treat analysis to test if baseline PET affected the subsequent 3-year peritonitis rate. Among all recruited PD patients, 308 (34.2%) developed peritonitis within three years. Multivariate Cox regression analysis showed that the high-transport group has the greatest peritonitis risk (HR 1.98, 95% CI: 1.08–3.62) even after an adjustment for demographics, comorbid diseases, and biochemical measurements. We concluded that a baseline high peritoneal membrane transport rate is an independent risk factor for peritonitis in incident PD patients.

## 1. Introduction

Chronic kidney disease is a rapidly increasing global health burden [1,2]. According to a systemic analysis, the global prevalence of chronic kidney disease is 9.1% and causes more than one million deaths every year [3]. This prevalence ranges from 3.3% in Norway to 14.8% in the United States [3]. Dialysis is a treatment used to remove waste products from the blood when the kidneys are not functioning properly. In hemodialysis (HD), the artificial dialyzer filters the blood through a membrane, and the waste products that pass through the membrane are washed away with dialysate. Peritoneal dialysis (PD) is another type of dialysis that does not require regular hospital visits, which provides a better quality of life [4,5]. The biggest difference between the two dialysis modalities is that PD uses the peritoneum as a filter [6]. In PD treatment, the dialysate flows through a catheter into the peritoneal cavity, and the peritoneum acts as a natural filter membrane to remove waste products. It has been recognized that PD patients have different initial peritoneal membrane transport characteristics. These differences are best classified and determined using the peritoneal equilibration test (PET), a semi-quantitative measurement of the peritoneal membrane transfer rate [7,8]. The peritoneal membrane transport classification is based on averages, standard deviations, and the minimum and maximum values of creatinine dialysate/plasma and glucose D/D0 in the fourth hour of dialysis [9]. The test allows the comparison of multiple results in a particular patient over a long period of therapy. Peritoneal membrane transport characteristics, however, change over time and tend to increase after peritonitis and long-term PD treatment [10]. It is impractical to use a random PET to predict the peritonitis rate in pre-existing PD patients, especially in those with recent peritonitis and long dialysis vintage.

Peritonitis is a common complication of PD that causes significant morbidity [11] and technical failure [12]. Various risk factors have been proposed as being associated with PD peritonitis, including old age [13], being female [14,15,16], diabetes mellitus [17], coronary artery disease [6], hypertension [18], and hypoalbuminemia [19,20]. Nevertheless, most of these studies present a limited cohort of patients, and there are contradictory results. In addition, some of these studies are based on registered data with large patient numbers [13,14,17] but none of them analyzed the characteristics of the peritoneal membrane, the first barrier to block potential microorganism invasion during PD dialysate exchange, which affects the peritonitis rate. In this study, we analyzed a 20-year PD cohort comprising 898 incident patients, to investigate whether there are unidentified causative factors that influence the PD peritonitis rate.

## 2. Materials and Methods

### 2.1. Study Design

In this study, incident peritoneal dialysis patients at the Taipei Veterans General Hospital from 1 January 1998 to 31 December 2019 were enrolled. Patients were categorized into four groups, based on their baseline PET D/P ratio, and were subsequently followed up for three years regarding peritonitis. Meanwhile, hemogram and serum biochemical data were collected monthly. Patients with missing PET profiles, hybrid hemodialysis, and peritoneal dialysis, and those with the wrong diagnostic code of end-stage kidney disease etiology were excluded from the study. Peritonitis events that were clearly caused by other reasons than peritoneal dialysis treatment were also excluded from the analysis. The Institute Review Board (IRB) reviewed and approved the study design (IRB No 2021–12-007C).

### 2.2. Peritoneal-Membrane Equilibration Test

Standard 4-hour PET tests with 2.5% dextrose dialysate were used in the current study. The solute transport rates across the peritoneal membrane were assessed according to equilibration rates between the peritoneal capillary blood and dialysate. The peritoneal transport rate was calculated using the ratio of creatinine concentration in dialysate and plasma (D/P ratio) at various time points. According to their peritoneal transport rate, patients were divided into four groups: high (4-h D/P ratio above 0.82), high–average (0.66–0.81), low–average (0.51–0.65), or low transporter (0.35–0.50) groups. In our hospital, all PD patients received their first peritoneal equilibration test between 1 and 3 months after the commencement of PD.

### 2.3. Diagnosis of Peritonitis

According to the 2016 ISPD guidelines [21], peritonitis was diagnosed when at least two out of the following conditions were present: (1) consistent clinical features, (2) dialysis effluent WBC > 100/μL and polymorphonuclear neutrophil > 50%, (3) a positive dialysate effluent culture. Patients with peritonitis before their first PET examination were excluded from the analysis.

### 2.4. Statistics

Statistical analysis was performed using the IBM IPSS statistics software, V22.0. We used the intention-to-treat model because the D/P ratio tends to change during PD treatment, and the increasing rate of the D/P ratio varies among individuals. The peritonitis rates of four groups were illustrated using a Kaplan–Meier curve and were analyzed with the log-rank test. To identify the independent risk factors of peritonitis, variables with a *p*-value of less than 0.1 in univariate analysis were put into a multivariate Cox regression model. All tests were two-tailed and a *p*-value of less than 0.05 was considered significant.

## 3. Results

### 3.1. Demographics of Study Cohort 

**Figure 1:**
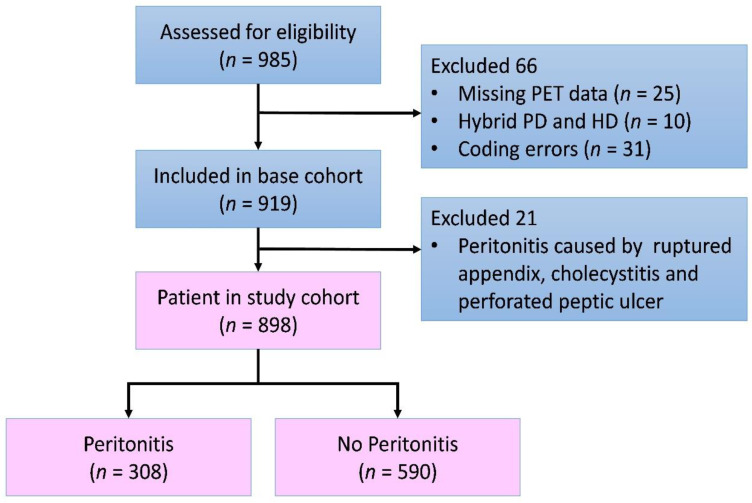
Flow chart of the study cohort. Abbreviations: PD—peritoneal dialysis; HD—hemodialysis.

There were 985 incident patients who began PD treatment in the recruiting period. After excluding those patients with missing data, coding errors, and peritonitis occurring before the first PET, 898 patients were recruited (Figure 1). Their mean age was 52 ± 15.4 years old; 46.2% were male, 41.9% had type I or type II diabetes, 3.7% had coronary artery disease, 5.2% had congestive heart failure, 1.7% had cirrhosis, and 90.3% of patients received continuous ambulatory peritoneal dialysis (CAPD). The mean plasma albumin was 3.7 g/dL. Based on the aforementioned first PET result, there were 117(13%) in the high group, 402 (44.8%) in the high–average group, 321(35.7%) in the low–average group, and 58 (6.5%) in the low transport group. The demographics and clinical variables are listed in Table 1.

### 3.2. Peritoneal Membrane Transport Characteristics and Peritonitis Rate 

**Figure 2:**
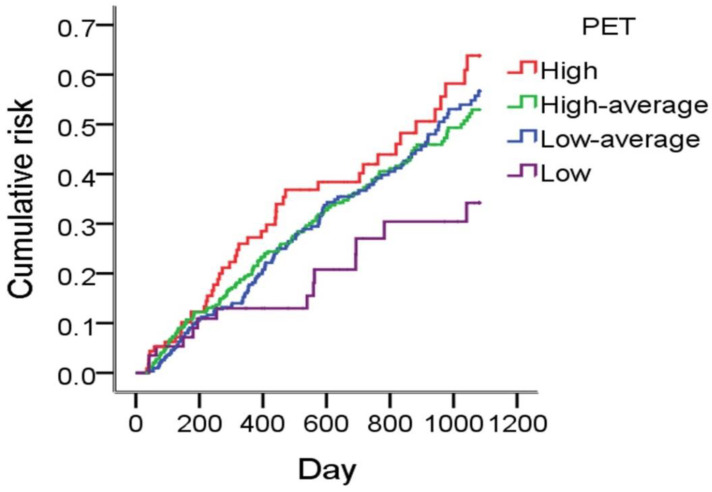
Peritonitis rates in different peritoneal transport groups.

After three years of follow-up, 308 (34.2%) patients had had peritonitis events. The average follow-up period was 814 days and the crude rate of peritonitis was 0.18 episodes/patient-year. The Kaplan–Meier plot illustrated that patients with different baseline peritoneal membrane characteristics had different peritonitis rates, while the low transporters were protected, and the high transporters had the highest rate (Figure 2). In the univariate Cox regression model, we identified PET, coronary artery disease, heart failure, hypertension, and cirrhosis as potential risk factors. In the multivariate Cox regression analysis, only high transporters (HR 1.98, 95% CI: 1.08–3.62, *p* = 0.026), coronary artery disease (HR 2.07, 95% CI: 1.23–3.49, *p* = 0.006), and cirrhosis (HR 3.40, 95% CI: 1.60–7.23, *p* = 0.002) were identified as independent risk factors for peritonitis. There was no significant difference in peritonitis risk between high–average (HR: 1.61, 95% CI: 0.93–2.81, *p* = 0.091), low–average (HR: 1.71, 95% CI: 0.98–2.99, *p* = 0.060) and low transporter groups. In our cohort, diabetes, congestive heart failure, hypertension, malignancy, and plasma albumin level were not associated with peritonitis risk (Table 2).

### 3.3. Peritoneal Membrane Transport Characteristics and Peritonitis Microbiology 

**Figure 3:**
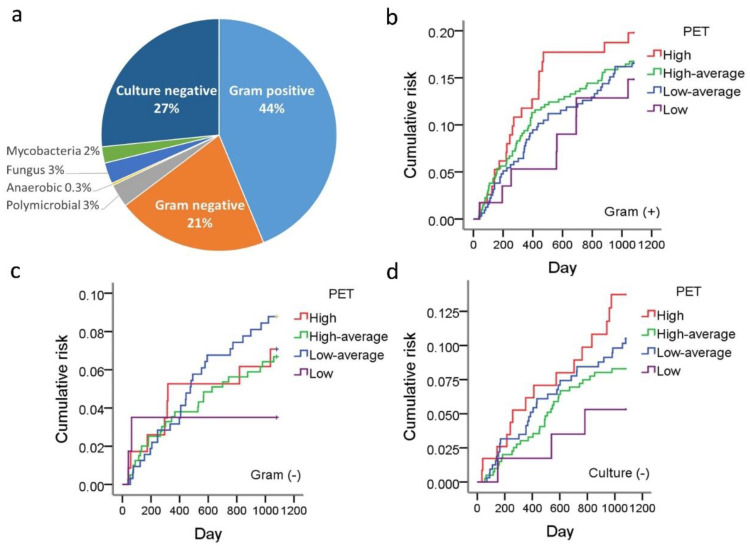
Subgroup analysis of peritonitis rates in different microbial etiologies. The percentage of different microbial etiologies is illustrated in panel (**a**). The peritonitis rates of the four transport statuses regarding different pathogenic microorganisms are illustrated as Gram-positive bacilli (**b**), Gram-negative bacilli (**c**), and culture-negative (**d**).

Of the 308 peritonitis episodes, 44% were caused by Gram-positive bacteria, whereas Gram-negative bacteria accounted for 21%, and 27% of cases were culture-negative. We analyzed the association between PET and the peritonitis rate in each microbiological category. There was a trend that high transporters showed an increased Gram-positive and culture-negative peritonitis rate, but neither of them reached statistical significance (Figure 3).

## 4. Discussion

In this study, we reported that the baseline D/P ratio is an independent risk factor of peritonitis for the first 3 years. As far as we were concerned, this was the biggest cohort employed for studying the association between PET and peritonitis. We discovered that rapid transporters have an 86% higher peritonitis risk than low transporters. This finding highlighted that in incident PD patients, PET not only characterized the solute transport rate but also reflected the peritonitis risk. Hence, nephrologists and PD nurses must pay more attention to peritonitis prevention when treating patients with a rapid peritoneal solute transport rate.

Peritoneal dialysis involves diffusive and convective transports and osmosis via the highly vascularized peritoneal membrane. With long-term exposure to uremic toxins and high-glucose PD dialysate, neo-angiogenesis and increased vascular density alter the peritoneum’s functions [22]. The peritoneal capillary endothelium offers a rate-limiting hindrance to solute and water transport [23]. Morphological studies have illustrated that peritoneal membrane vascular density and vasculopathy increased with the duration of PD [24], and that PD patients usually experienced a D/P ratio increase before they lost ultrafiltration capability [25]. Previous studies also showed that rapid transporters had a higher risk of ultrafiltration failure, technique failure [26], and mortality [27]. For the first time, our data here has revealed that these patients also carried a higher risk of peritonitis. 

Only a few studies have analyzed the association between PET and subsequent peritonitis. Sarah So et al. [28] analyzed 397 PD patients and concluded that baseline PET could not predict the 10-year peritonitis rate. The D/P ratio did increase in some, but not all patients, after long-term PD treatment; therefore, the rate of peritoneal dysfunction varies widely among individuals. It is therefore difficult to believe that an initial PET would affect the long-term peritonitis rate. A study published by a group in Spain provided baseline demographic data, including the peritoneal D/P ratios of 565 PD patients, but the aim of that study was to find a predisposing factor of PD peritonitis mortality rather than assessing infection risk [15]. In the current study, we used intention-to-treat analysis to study the baseline PET categories, along with the 3-year peritonitis rate among 898 incident PD patients. Our data showed that patients with rapid peritoneal solute transport had the highest peritonitis rate, whereas patients with a low transport rate had the lowest risk. This finding demonstrated that baseline PET not only characterized the peritoneum solute transport rate but also reflected the risk of peritonitis.

In our cohort, patients from four PET categories had similar peritonitis rates until 1 year after PD initiation, which suggested that peritoneal transport characteristics did not influence the early-onset peritonitis rate. Previous studies have reported catheter exit-site infection [29], nasal staphylococcus aureus carriers [30], and patient training [31,32,33] as risk factors for early-onset peritonitis [29]. The fact that different PET groups had comparable peritonitis rates in the first year suggested that the higher peritonitis rate in rapid transporters was not caused by contamination during PD dialysate exchanges but was instead caused by compromised peritoneal immunity. Since rapid transporters carried a higher risk of mortality and technique failure, it was hypothesized that a rapid peritoneal transport rate is a consequence of systemic inflammation. However, new evidence has shown that the D/P ratio was positively associated with dialysate effluent inflammatory markers, such as interleukin 6 and TGF-β [34,35], but not with serum inflammation markers [34,35,36], indicating that a high D/P ratio represents intraperitoneal rather than systemic inflammation [37]. As a consequence, patients with a rapid solute transport rate usually have a high vascular density, impaired endothelial integrity [22,38,39], and greater dialysate protein loss [40,41,42]. It was possible that this intra-abdominal inflammation also altered local immunity response, which made the high transporters more prone to developing PD-related peritonitis. The fact that cirrhosis patients have a higher proportion of rapid transporters, and an increased peritonitis rate supports our hypothesis that peritoneal inflammation is a predisposing factor of peritonitis.

Coronary artery disease was also an independent risk factor of PD-related peritonitis in our study. McDonald et al. [13] reported the same finding in their study. The mechanism behind this association remains unclear. Diabetes, hypertension, and smoking are risk factors for coronary artery disease and have been reported as a peritonitis risk factor in some but not all studies [43]. We did not observe these associations in our data. 

There are several inherited limitations to this study. Because this is a retrospective observational study, potential causative factors and confounders could have been missed. Potential causative factors, like nasal carriages of *Staphylococcus*, smoking, and low socioeconomic status, were not available in our data. We also do not have data regarding solute clearance, drainage volume, PD dialysate prescription, and residual renal function.

## 5. Conclusions

Peritonitis is an important and severe complication among patients undergoing peritoneal dialysis. Various risk factors have been linked to the use of PD. We found that a high peritoneal transport status, liver cirrhosis, and coronary artery disease are independent risk factors of peritonitis in incident PD patients. We propose, for the first time ever, that high peritoneal transport status is associated with increased peritonitis risk, as identified in this study, although the mechanism remains unclear. The findings emphasize that nephrologists and PD nurses need to pay more attention to peritonitis prophylaxis in these patients.

## Figures and Tables

**Table 1:** Demographics and clinical variables, according to PET status.

Variable	Overall	H	HA	LA	L
Patients (*n*)	898	117	402	321	58
Age (%) <20 20–39 40–59 60–79 ≧80	0.9 21.5 43.9 30.5 3.2	0 17.9 39.3 39.4 3.4	1.0 22.6 43.8 28.1 4.5	0.6 20.9 47.4 29.3 1.8	3.4 24.1 34.5 36.2 1.8
Gender (% men)	46.2	57.3	49.8	40.2	32.8
Diabetes mellitus (%)	41.9	56.4	43.0	34.9	43.1
Hypertension (%)	63.0	63.2	63.2	64.2	55.2
Coronary artery disease (%)	3.6	2.6	3.7	3.4	6.9
Congestive heart failure (%)	5.2	3.4	6.5	4.7	3.4
Cirrhosis (%)	1.7	5.1	1.7	0.3	1.7
Malignancy (%)	2.0	4.3	1.7	1.6	1.7
Modality at initiation of PD (% CAPD)	90.3	85.5	91.0	91.0	91.4
Serum albumin (g/dL)	3.7 ± 0.6	3.7 ± 0.6	3.7 ± 0.6	3.7 ± 0.6	3.7 ± 0.6

Abbreviations: H, high; HA, high average; LA, low average; L, low transporters; PD, peritoneal dialysis; CAPD, continuous ambulatory peritoneal dialysis; SD, standard deviation.

**Table 2:** Uni-and multivariate Cox regression analysis of the peritonitis risk factor.

Variable	Univariate Analysis			Multivariate Analysis		
	Hazard Ratio	95% CI	*p*-Value	Hazard Ratio	95% CI	*p*-Value
PET L	Reference	-	-	Reference	-	-
LA	1.72	0.99–3.02	0.057	1.71	0.98–2.99	0.060
HA	1.59	0.91–2.76	0.105	1.61	0.93–2.81	0.091
H	1.90	1.04–3.50	0.038	1.98	1.08–3.62	0.026
Coronary artery disease	1.95	1.15–3.32	0.013	2.07	1.23–3.49	0.006
Congestive heart failure	1.50	0.94–2.38	0.086	1.55	0.98–2.46	0.063
Hypertension	0.80	0.63–1.01	0.056	0.84	0.66–1.06	0.131
Cirrhosis	3.46	1.61–7.42	0.001	3.40	1.60–7.23	0.002
Age	1.01	1.00–1.01	0.211			
Male gender	1.13	0.89–1.42	0.315			
Diabetes Mellitus	1.04	0.82–1.32	0.766			
Malignancy	0.71	0.26–1.91	0.496			
CAPD	1.36	0.87–2.13	0.175			
Plasma albumin	0.80	0.61–1.05	0.105			

Abbreviations: H, high; HA, high average; LA, low average; L, low; CAPD, continuous ambulatory peritoneal dialysis.

## Data Availability

Not applicable.

## Abbreviations

PET	Peritoneal equilibration test
PD	PD Peritoneal dialysis
HR	HR hazard ratio
HR hazard ratio	HR hazard ratio
CI	Confidence interval
HD	Hemodialysis
IRB	Institute Review Board
WBC	White blood cells
H	High
HA	High average
LA	Low average
L	Low
CAPD	Continuous ambulatory peritoneal dialysis
